# The Effects of Curtain Airbag on Occupant Kinematics and Injury Index in Rollover Crash

**DOI:** 10.1155/2018/4980413

**Published:** 2018-03-22

**Authors:** Hongyun Li, Chengyue Jiang, Dong Cui, Shuang Lu

**Affiliations:** ^1^China Automotive Technology and Research Center, Tianjin 300300, China; ^2^Key Laboratory of Advanced Manufacture Technology for Automobile Parts, Ministry of Education, Chongqing University of Technology, Chongqing 400054, China

## Abstract

**Background:**

Occupant injuries in rollover crashes are associated with vehicle structural performance, as well as the restraint system design. For a better understanding of the occupant kinematics and injury index in certain rollover crash, it is essential to carry out dynamic vehicle rollover simulation with dummy included.

**Objective:**

This study focused on effects of curtain airbag (CAB) parameters on occupant kinematics and injury indexes in a rollover crash. Besides, optimized parameters of the CAB were proposed for the purpose of decreasing the occupant injuries in such rollover scenario.

**Method and Material:**

The vehicle motion from the physical test was introduced as the input for the numerical simulation, and the 50% Hybrid III dummy model from the MADYMO database was imported into a simulation model. The restraint system, including a validated CAB module, was introduced for occupant kinematics simulation and injury evaluation. TTF setting, maximum inflator pressure, and protection area of the CAB were analysed.

**Results:**

After introducing the curtain airbag, the maximum head acceleration was reduced from 91.60 g to 49.52 g, and the neck M_x_ and neck F_z_ were reduced significantly. Among these CAB parameters, the TTF setting had the largest effect on the head acceleration which could reduce 8.6 g furthermore after optimization. The neck F_z_ was decreased from 3766.48 N to 2571.77 N after optimization of CAB protection area.

**Conclusions:**

Avoiding hard contact is critical for the occupant protection in the rollover crashes. The simulation results indicated that occupant kinematics and certain injury indexes were improved with the help of CAB in such rollover scenario. Appropriate TTF setting and inflator selection could benefit occupant kinematics and injury indexes. Besides, it was advised to optimize the curtain airbag thickness around the head contact area to improve head and neck injury indexes.

## 1. Introduction

Among all types of vehicle accidents, rollover crashes are the most complex and least understood [1]. Rollover crashes are associated with approximately 20% of all fatal crashes in the United States [2]. While in China, the rollover crashes constitute about 2.7% of all crashes but account for approximately 5.6% of the fatal crashes [3]. In recent years, Chinese automakers continue to gain share in the domestic market, especially in the sport utility vehicle (SUV) segment. According to statistics, SUVs accounted for 36% in all sales of Chinese passenger vehicle market in 2016 [4]. However, SUVs are more likely to suffer the rollover crashes due to their higher rollover stability factor, which is related to the track width and the height of the centre of gravity. Thus, it is important to understand certain SUV's rollover characteristics and the interaction between occupants and vehicle compartment.

Although the rollover crash causes high fatalities relative to its occurrence, there is no repeatable standardized dynamic rollover test procedure due to the chaotic nature of rollover crash [5]. Some previous physical tests and computer simulation studies were carried out to understand the mechanisms of such crash. In the research of Grzebieta et al. [6], it was concluded that to date the current test protocols were not capable of consistently replicating the injuries identified in real-world rollover crashes. Heller et al. [7] carried out two rollover tests utilizing instrumented test vehicles and instrumented ATDs to investigate occupant kinematics and injury response throughout the entire rollover sequences, in which the occupant's upper neck compressive loading was more than 4000 N in the sedan due to the head-to-vehicle and head-to-ground contact. Han and Seo [8] developed a finite element-based numerical vehicle simulation model consisting of a rigid lower body and a deformable upper body and addressed essential factors to improve the rollover performance. Jiang et al. [9] demonstrated the applicability of occupant kinematics simulation and head injury analysis with MADYMO simulation for rollover cases, including two real-world rollover crashes, together with an SAEJ2114 rollover test, which concluded that more real-world rollover crashes need replicating and simulating. Recent studies of restrained occupants involved in single-vehicle pure rollover crashes that occurred in the United States by Mattos et al. [10] and Bambach et al. [11] indicated that serious injuries to the thorax, head, and spine could still occur even when there is little or no roof crash, highlighting the need to improve occupant safety systems.

Improving the occupant safety in rollover is challenging due to the different types of rollover crashes. The rollover curtain airbag (CAB) was first introduced in the United States automotive market in 2002 for the purpose of providing incremental benefit to belted occupants in rollover crashes [12]. It is known from crash testing and data analysis that the curtain airbags provide considerable benefit to occupants in rollover crashes [12, 13].

For more detailed analysis, it is critical to perform computer simulations to understand the interactions between belted occupants and curtain airbags during a certain SUV rollover crash scenario. In this paper, a multibody dynamic rollover model was developed according to a rollover test. After validation, the effects of certain curtain airbag parameters on occupant protection performance in such rollover crash were evaluated.

## 2. Method

This study focused on the simulation of vehicle dynamic rollover, as well as the assessment of curtain airbag parameters. A multibody rollover simulation model, including rigid vehicle chassis and occupant compartment model, was built and validated with a dynamic rollover test from the University of Virginia. Based on the correlated vehicle model, the 50% Hybrid III dummy model from the MADYMO database [14] was imported for the occupant kinematics simulation.

To evaluate the effects of CAB on both occupant kinematics and injury index, a validated CAB module from side impact was included in the simulation model for further injury evaluation. The effects of certain CAB parameters, including TTF setting, inflator pressure, and CAB protection area, upon occupant protection performance, were evaluated.

### 2.1. Rollover Test and Modeling

**Figure 1:**
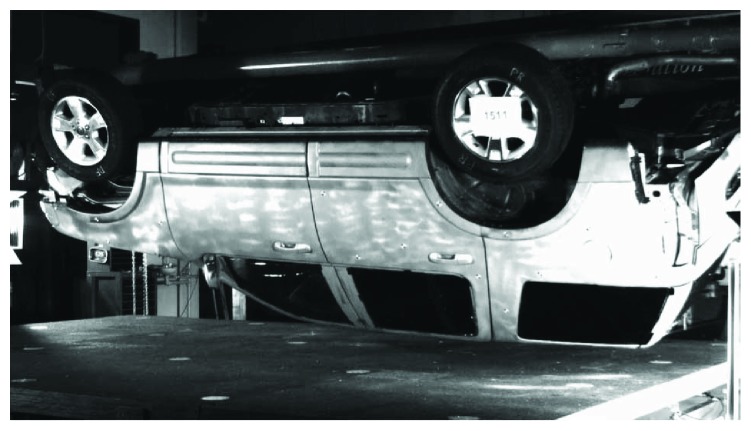
Rollover test at the moment of vehicle to ground contact.

So far, the dynamic rollover test system in the University of Virginia [15] is one of the test rigs that may be capable of repeatable rollover test. For the following vehicle rollover simulation, a dynamic rollover test carried out by the University of Virginia was introduced in this study. In this rollover test, the vehicle rotational motion and drop height were controlled. The scenario of the rollover test is shown in Figure 1.

**Figure 2:**
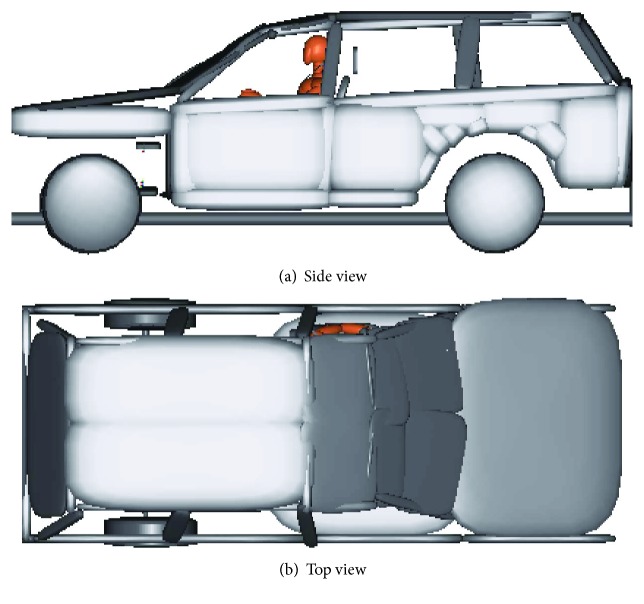
Rollover simulation model with Hybrid III dummy model.

The original vehicle FE model used for modeling in this paper was developed at the National Crash Analysis Center (NCAC) [16], which was validated to several subsystem tests and two FMVSS No. 216 quasistatic tests. To reduce the computation time, a multibody rollover simulation model, including rigid vehicle chassis and occupant compartment, was built for the following analysis. The major components of the vehicle model were positioned according to the vehicle FE model. A 50% Hybrid III dummy model from the MADYMO database was imported for the occupant kinematics simulation and injury evaluation. The occupant model was belted with both shoulder belt and lap belt. The whole simulation model is shown in Figure 2.

The initial settings of the physical rollover test were introduced as the input and boundary conditions for the numerical simulation. The vehicle motion in the simulation was defined by prescribing the motion for a free joint at the vehicle center of gravity (COG) with all degrees of freedom. The restraints for the vehicle, as the main parameters affecting the vehicle rollover simulation results, were set according to the test information. The initial roll velocity was 190°/s, and the test bed speed was 24 km/h.

### 2.2. Rollover Model Validation

**Figure 3:**
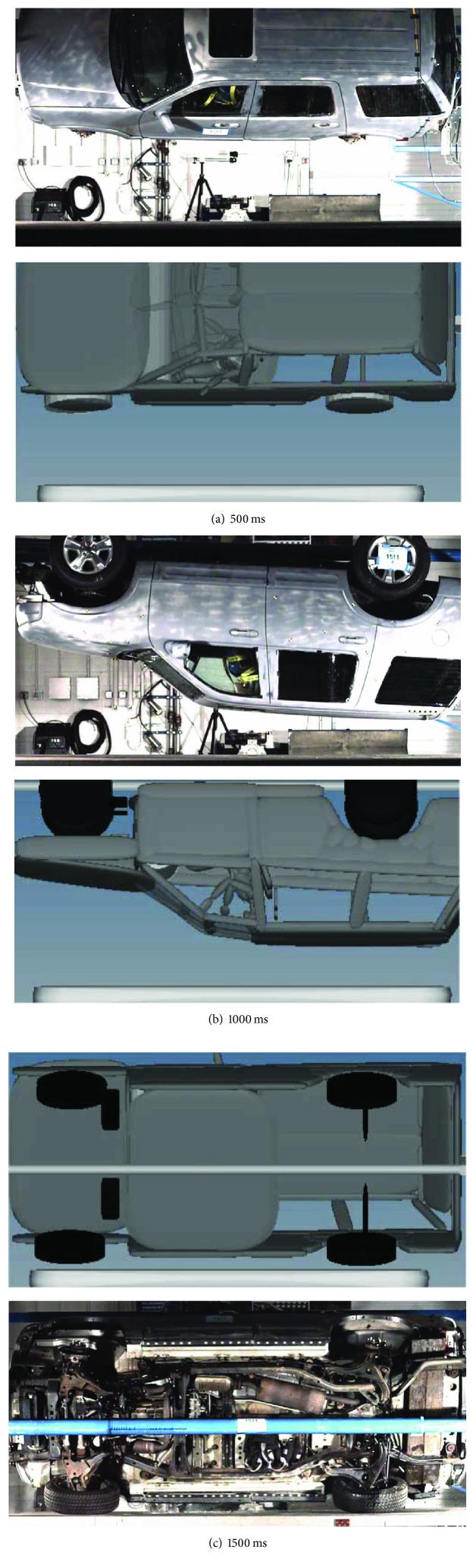
Vehicle rollover trajectory correlation.

The validation of rollover simulation model was carried out by comparing the vehicle trajectory from the simulation with that of the test. As shown in Figure 3, the vehicle trajectory of simulation correlated well with that of the rollover test. The simulation result indicated that the vehicle roof made contact with the test bed at similar angle with that of the test.

### 2.3. Occupant Kinematics Simulation

To simulate the occupant kinematics in such rollover scenario, a 50% Hybrid III dummy model from the MADYMO database was placed at the driver position. The occupant compartment was built according to the geometry of the vehicle, which included instrument panel, seat, and FE seat belts. The component contact stiffness was mainly from the component test and MADYMO example case, as in the research of [17].

**Figure 4:**
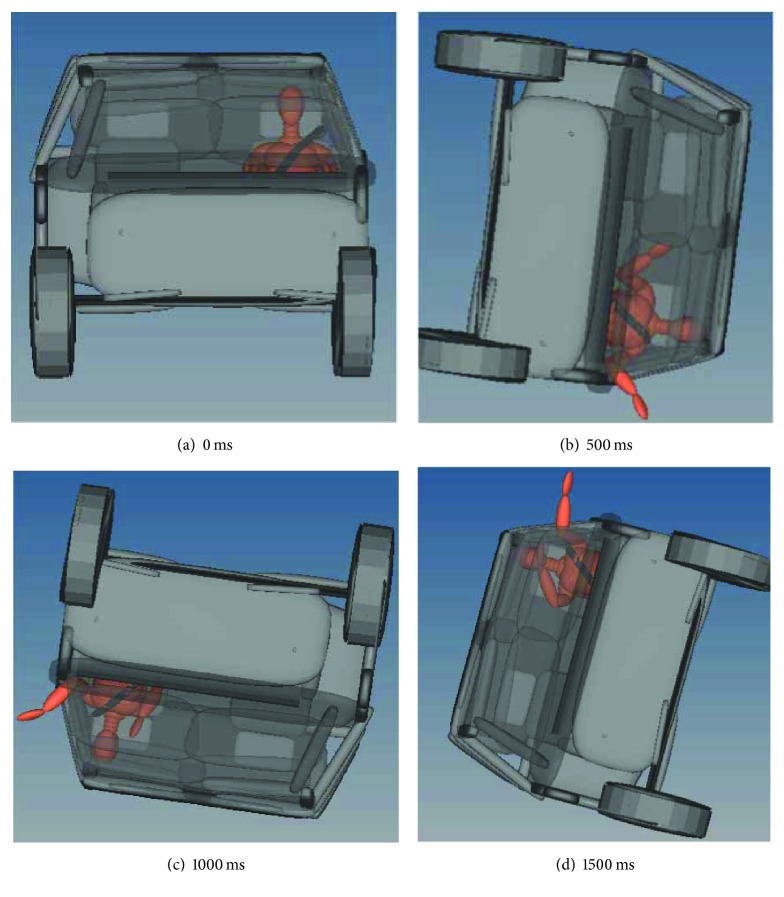
Occupant kinematics in rollover simulation.

The occupant kinematics of the simulation from 0 ms to 1500 ms was shown in Figure 4. It was found that the left arm of the occupant was ejected around 300 ms. Besides, the occupant's head made contact with the vehicle interior around 1090 ms. This was similar to the finding of Huelke et al. [18], who indicated that 33% head injuries resulted from contact to vehicle interior surfaces. Furthermore, the contact between the head and vehicle resulted in the 5892.44 N neck F_z_, which corresponded to the research result of Heller et al. [7]. Heller et al. found that the occupant's upper neck compressive loading was more than 4000 N in the sedan due to the head-to-vehicle contact.

Thus, further attention was paid on how to reduce such hard contact and improve the injury index in the following study.

### 2.4. Curtain Airbag (CAB) Model

The curtain airbag was firstly invented to protect the occupants during side impacts and rollover crashes. In case of the rollover crashes, introducing the curtain airbag could help mitigate the occupant ejection [19, 20].

**Figure 5:**
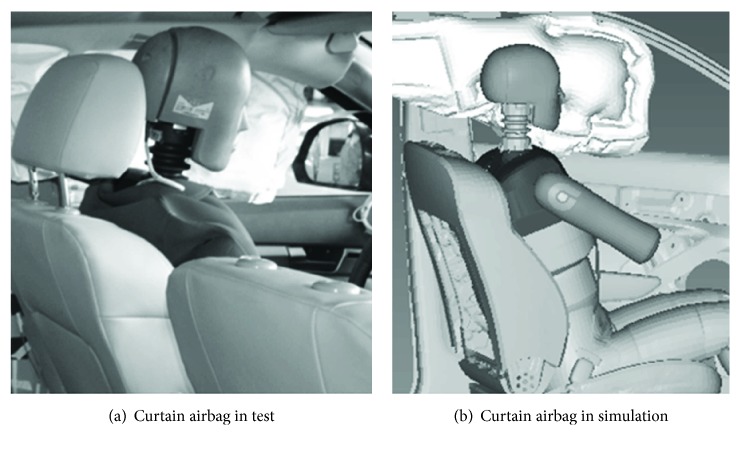
Curtain airbag in the test and simulation.

The main objective of this study is to evaluate the effects of curtain airbag on occupant kinematics and injury indexes. Thus, a curtain airbag which was validated in certain side impact simulation was imported for further analysis in this study, as shown in Figure 5. The curtain airbag geometry and inflator data were from the supplier.

To analyze the effects of curtain airbag on occupant kinematics and injury index, the CAB model above was imported to the rollover simulation model and installed around the roof rail area.

## 3. Simulation Results and Injury Analysis

### 3.1. Occupant Kinematics with CAB

**Figure 6:**
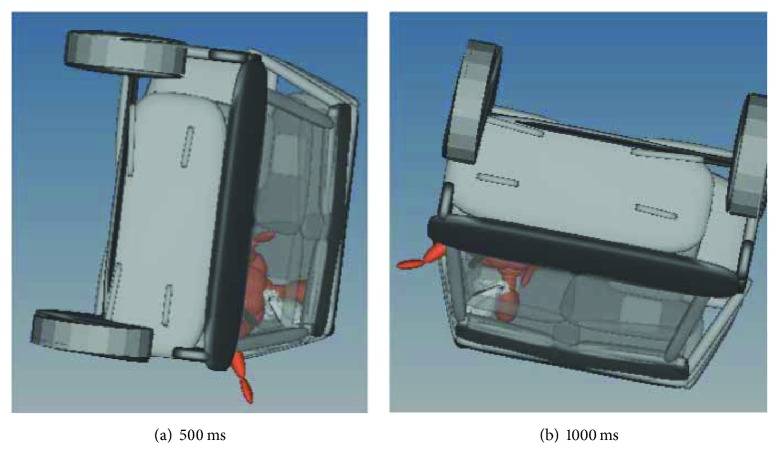
Occupant kinematics with curtain airbag in rollover simulation.

After introducing the curtain airbag, the occupant kinematics at 500 ms and 1000 ms was shown in Figure 6.

From Figure 6, it was found that introducing the curtain airbag could help to keep the head of the occupant in the passenger compartment during the whole rollover simulation, and the contact force between the head and vehicle interior was decreased. However, the left arm of the occupant was still ejected around 300 ms.

### 3.2. Occupant Injuries with CAB

**Figure 7:**
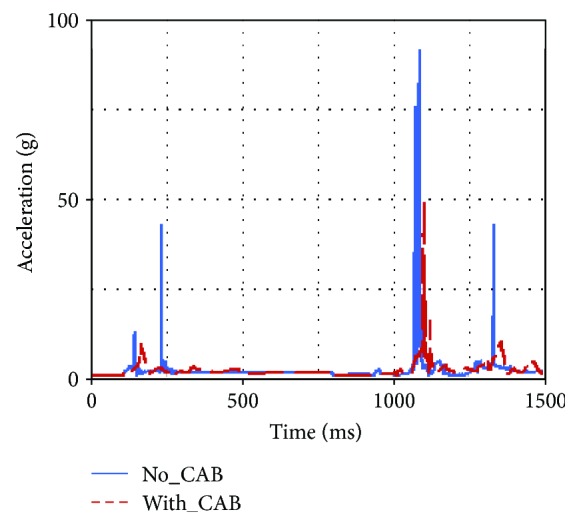
Head resultant accelerations.

**Figure 8:**
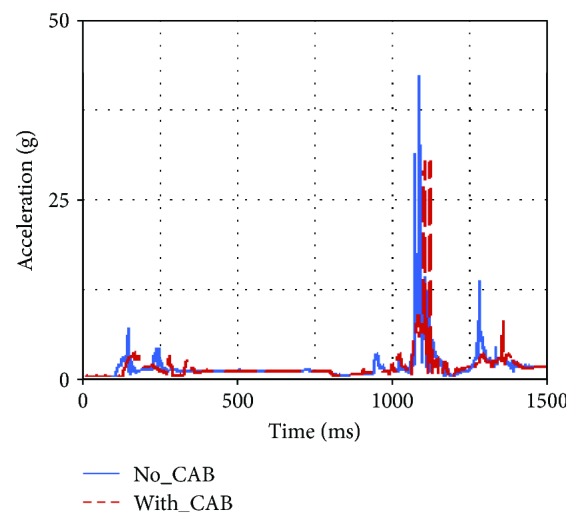
Chest resultant accelerations.

**Figure 9:**
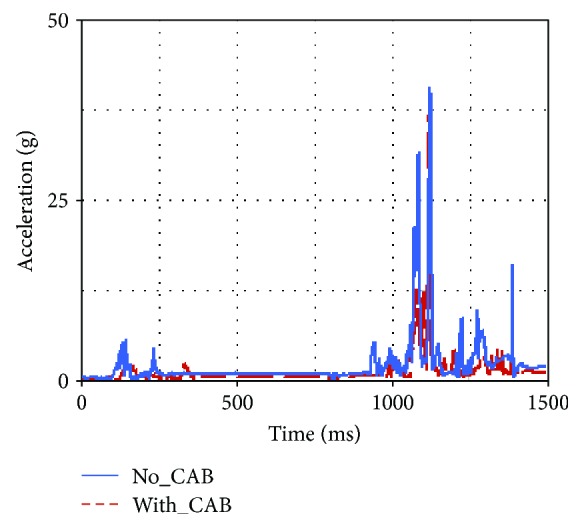
Pelvis resultant accelerations.

**Figure 10:**
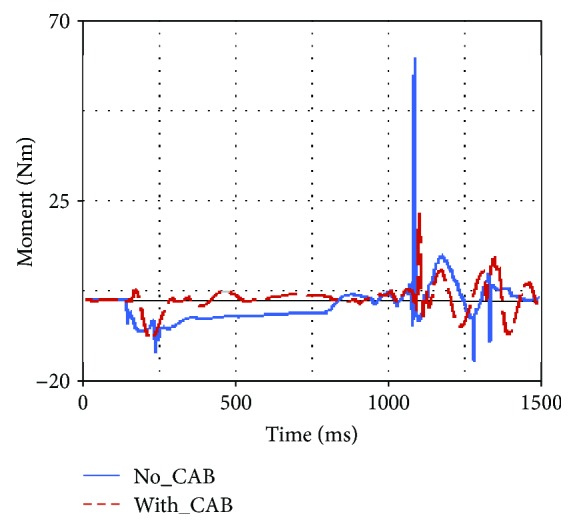
Upper neck M_x_.

**Figure 11:**
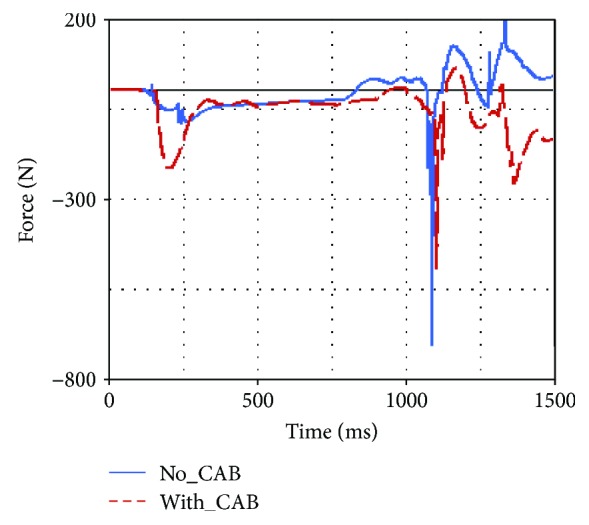
Upper neck F_y_.

**Figure 12:**
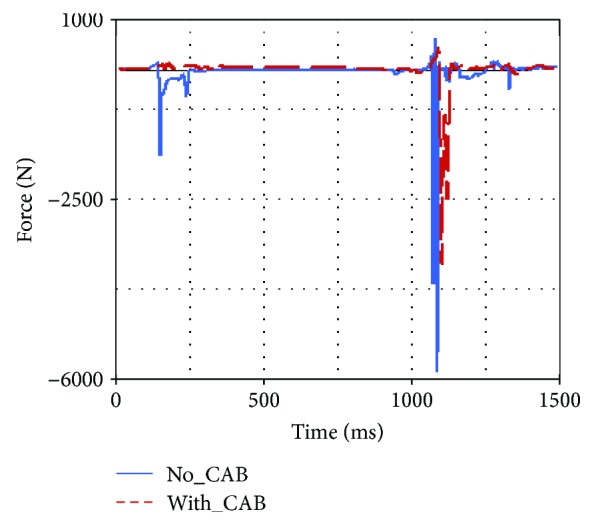
Upper neck F_z_.

Occupant injuries of both With-CAB case and No-CAB case were shown in Figures 7–12. The injury index curve of the No-CAB case is the blue line, while the curve of the With-CAB case is the red line.

As shown in Figures 7–9, both head resultant acceleration and chest resultant acceleration were reduced when the CAB was applied in vehicle during such rollover simulation, while the pelvis resultant acceleration was almost the same. It was obvious that the CAB mainly affected the head and chest regions, and it had little influence on the pelvis. This finding was similar to the real-world rollover crash studies [21]. The upper neck injury indexes, including the neck M_x_, neck F_y_, and neck F_z_ (Figures 10–12), were all improved. The maximum values of the neck M_x_ and neck F_z_ of the With-CAB case were reduced significantly.

**Figure 13:**
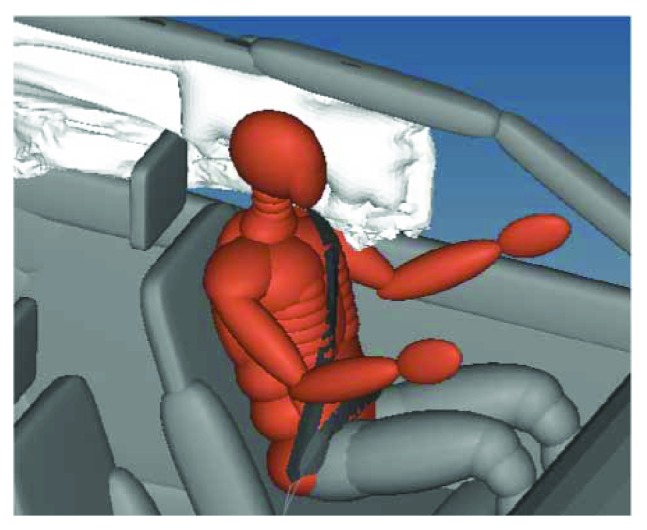
Contact between the head and curtain airbag at 225 ms.

After introducing the curtain airbag, the head's relative displacement toward B-pillar was reduced and its hard contact with B-pillar/roof rail could be avoided as well, as shown in Figure 13. This could also explain the reduction of head resultant acceleration for the With-CAB case around 225 ms, as shown in Figure 8.

In addition, the simulation result showed that the head's position remained around the medium position relative to the headrest with the support of CAB, and the distance between the head to the vehicle interior was increased, which was beneficial for the contact force reduction between the head and the interior. This explained that the head acceleration at 1090 ms was decreased dramatically for the With-CAB case.

### 3.3. The Influence of CAB Time to Fire (TTF) on Occupant Injuries

According to the kinematics of occupant in the No-CAB case, it was found that there was a contact between the head and B-pillar/roof at 225 ms. Usually, the CAB inflation time was about 35 ms. The ideal CAB TTF was that the head made contact with the CAB after it was full deployed. Thus, the CAB TTF was advised to set before 190 ms. Considering the margin value, TTF earlier than 180 ms was suggested.

After the TTF estimation above, three simulation cases with different CAB TTF settings were designed to evaluate such parameter's effects upon occupant injuries. The simulation results were listed in Table 1.

**Figure 14:**
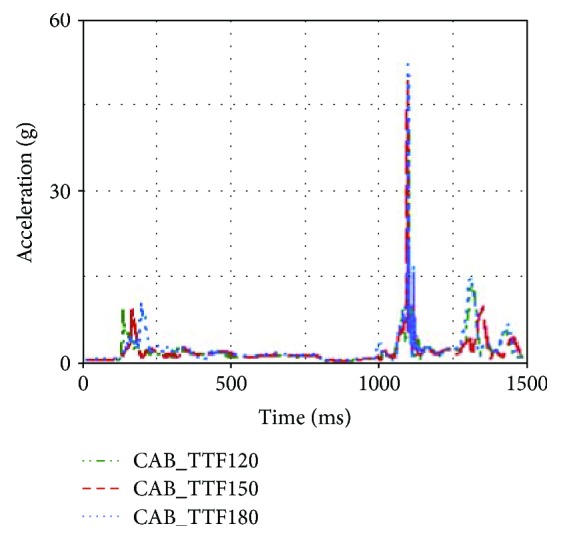
Head accelerations of different TTFs.

From Table 1, it was found that head acceleration and chest acceleration were increased when CAB TTF was increased from 120 ms to 180 ms. Besides, the neck injury indexes were also increased. This was because the head displacement toward the B-pillar was reduced due to earlier CAB TTF. In the rollover, an earlier CAB TTF setting could result in earlier contact between the head and CAB. Thus, an earlier CAB TTF setting could make the head acceleration of the first contact occur earlier, as shown in Figure 14.

**Figure 15:**
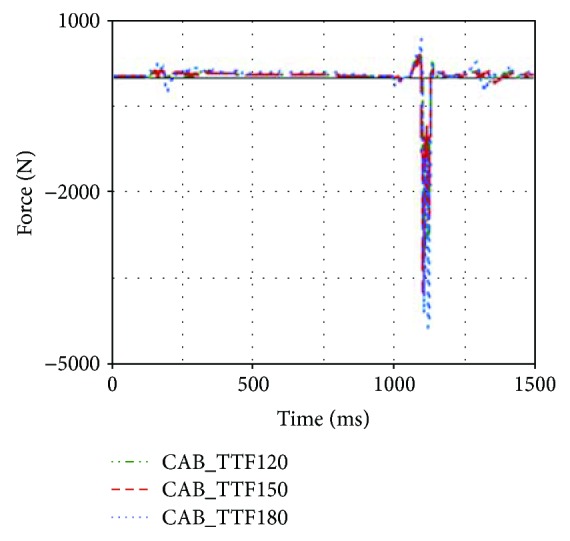
Upper neck F_z_ of different TTFs.

Earlier contact between the head and CAB would affect the neck injury indexes as well. Compared with that of CASE2, the upper neck F_z_ of CASE1 was reduced by 659.41 N, as shown in Figure 15.

### 3.4. The Influence of CAB Inflator Pressure on Occupant Injuries

The maximum pressure of inflator is a key factor for the CAB design. Three inflators with different maximum pressure were introduced in the simulation model for the following analysis. The simulation results were listed in Table 2.

According to Table 2, head acceleration, chest acceleration, and neck F_z_ were reduced when the maximum pressure of CAB inflator was increased from 220 kPa to 260 kPa. By contrast, neck F_y_ and neck M_x_ were increased when the maximum pressure of CAB inflator was increased.

**Figure 16:**
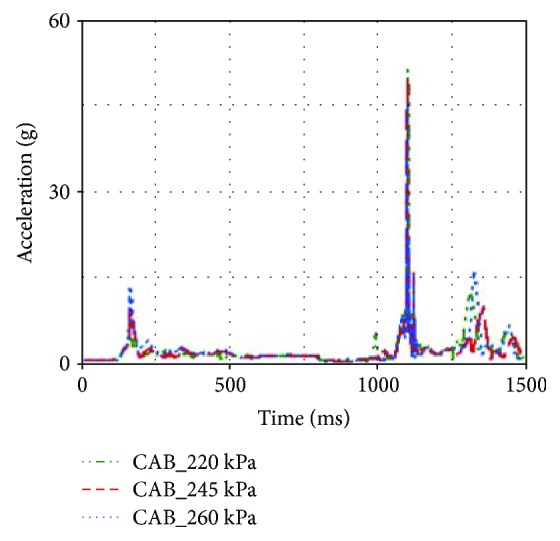
Head accelerations of different inflators.

**Figure 17:**
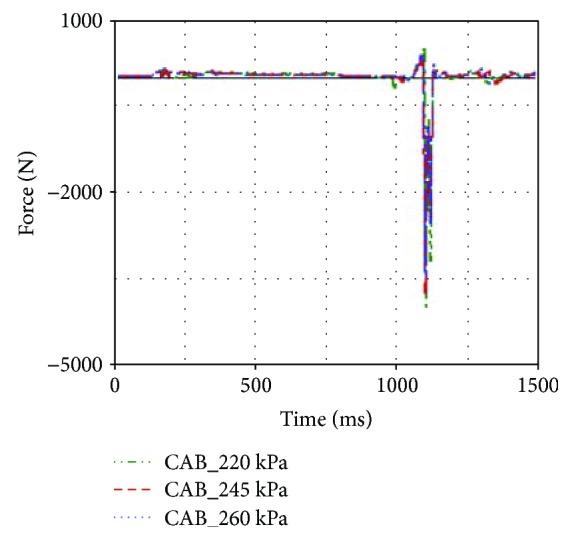
Upper neck F_z_ of different inflators.

In general, the increase of CAB inflator pressure could result in more support to the occupant and larger contact force between the head and CAB. In this rollover case, the neck F_y_ and neck M_x_ were increased when the 260 kPa inflator was used. However, larger inflator made the head and chest regions move away from the B-pillar. The accelerations of the head and chest were reduced (see Figure 16), while the neck F_z_ was also reduced when larger inflator was applied in the simulation model (see Figure 17).

### 3.5. The Influence of CAB Protection Area on Occupant Injuries

**Figure 18:**
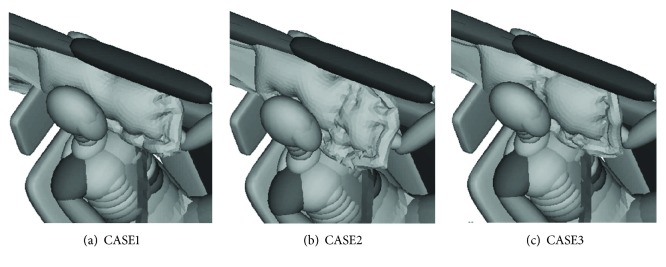
Three types of CAB with different protection areas.

Three types of CAB design with different protection areas were proposed to analyze the influence of the CAB protection area on occupant injuries, as shown in Figure 18.

CASE1 was the original CAB design. CASE2 was modified from CASE1, in which the thickness of frontal areas was reduced by the airbag suture. In CASE2, the position of airbag suture was 150 mm far from the head contact area. While for CASE3, the position of airbag suture was in the area of the head contact.

Three types of CAB were introduced to simulation model, and the simulation results were shown in Table 3.

**Figure 19:**
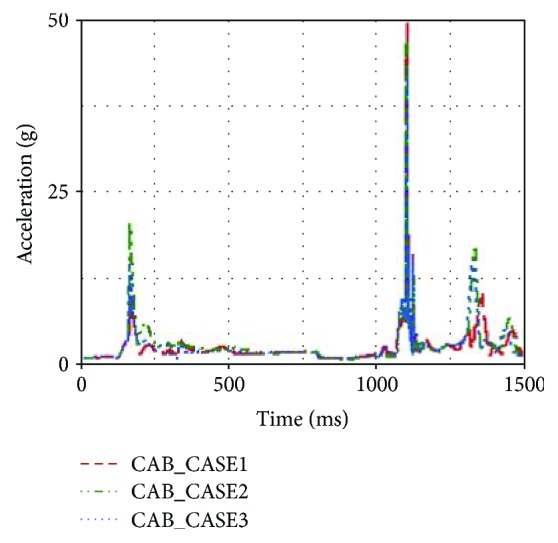
Head accelerations of different designs.

**Figure 20:**
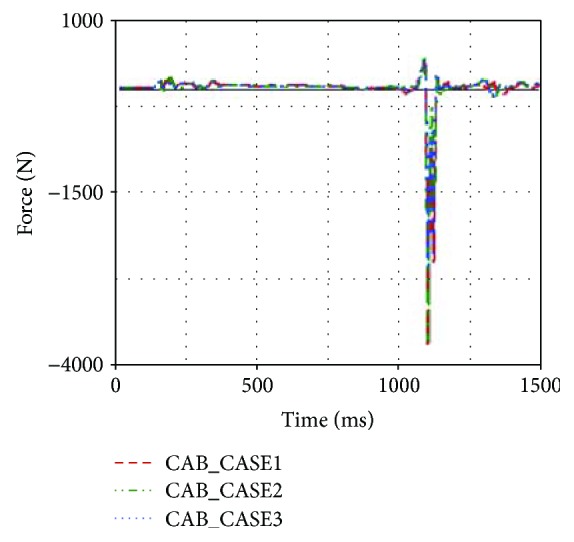
Upper neck F_z_ of different designs.

It was found that the head acceleration was improved when the CASE3 CAB was used (see Figure 19). Neck injury indexes for the CASE3 were reduced as well, especially for the neck F_z_, which was reduced by 1194.71 N (see Figure 20).

From the above analysis, it could be seen that the CAB design parameters, including the TTF setting, inflator data, and protection area (or airbag thickness), are critical for the improvement of occupant kinematics and injury indexes in such rollover crash, which need detailed concern for certain vehicle restraint system design.

## 4. Discussions

The effects of CAB on occupant injuries were mainly on the occupant head region, as well as the neck and thorax regions. After introducing a validated CAB module, the maximum head resultant acceleration was reduced from 91.60 g to 49.52 g. This was not only because CAB served as an energy absorbing object, but also it improved the occupant's upregion kinematics. As shown in Figure 13, the head's relative displacement toward B-pillar was reduced and its hard contact with B-pillar/roof rail was avoided as well in this case. Avoiding hard contact is critical for the occupant protection in the rollover crashes, which was pointed out in the research of [11] as well. Introducing CAB can help reduce the head acceleration and, therefore, decrease the head injury possibilities as in the research of [22].

For the influence of CAB TTFs on occupant injuries, it was found that the earlier TTF setting (between 120 ms to 180 ms) could reduce occupant injuries. However, TTF setting has to cooperate with rollover sensor calibration as well as the pressure holding capability of the CAB, since the CAB pressure powered by a hybrid inflator usually reaches its peak around 40 ms and starts to decay afterwards [23].

In the actual design process, study on the inflator selection needs to be conducted for the balanced curtain airbag design. The maximum pressure of the inflator and pressure holding capability of the CAB need to be considered for both system and the component requirements.

Except the CAB TTF setting, pressure of the inflator, and protection area, there are still some other parameters that need to be optimized in further study, such as the pressure holding capability and CAB tether, as well as the friction coefficients of airbag fabric [13]. All these parameters need to be analyzed in the follow-up studies.

## 5. Conclusions

In this study, a multibody rollover simulation model of SUV was developed according to the physical test. The vehicle kinematics from the simulation correlated very well with that of the rollover test. Based on the validated model, a validated curtain airbag was imported and its main parameters' effects on occupant kinematics and injury indexes were evaluated.

Upon the above analysis, the following conclusions could be drawn:

- In this rollover case study, the main injury source for the case without curtain airbag was the contact with the vehicle interior. Introducing the curtain airbag could improve the occupant kinematics and upbody injury indexes, although the left arm was ejected in this case.
- CAB design parameters, including the TTF setting, inflator data, and protection area, could affect certain injury indexes significantly. Among which, the TTF setting had the largest effect on the head acceleration. The maximum head acceleration was reduced 8.6 g after changing the TTF from 180 ms to 120 ms. The neck F_z_ could be decreased from 3766.48 N to 2571.77 N after the optimization of CAB protection area.
- For this rollover case study, it was advised to optimize the curtain airbag thickness around the head contact area, for the reduction of the head and neck injury indexes.

## Figures and Tables

**Table 1:** Occupant injury indexes with different CAB TTFs.

Number	CAB TTF (ms)	Head acceleration (g)	Chest acceleration (g)	Neck F_y_ (N)	Neck F_z_ (N)	Neck M_x_ (Nm)
CASE1	120	40.91	25.39	−408.44	−3071.07	14.19
CASE2	150	49.52	30.51	−492.57	−3766.48	22.08
CASE3	180	52.34	31.69	−522.54	−4382.55	28.73

**Table 2:** Occupant injuries with different airbag inflators.

Number	Maximum pressure (kPa)	Head acceleration (g)	Chest acceleration (g)	Neck F_y_ (N)	Neck F_z_ (N)	Neck M_x_ (Nm)
CASE1	220	51.44	33.28	−472.71	−4006.11	18.82
CASE2	245	49.52	30.51	−492.57	−3766.48	22.08
CASE3	260	46.73	29.43	−541.78	−3467.94	26.13

**Table 3:** Occupant injuries with three types of CAB.

Number	Head acceleration (g)	Chest acceleration (g)	Neck F_y_ (N)	Neck F_z_ (N)	Neck M_x_ (Nm)
CASE1	49.52	30.51	−492.57	−3766.48	22.08
CASE2	47.29	29.46	−423.21	−3682.75	21.83
CASE3	42.67	25.28	−414.56	−2571.77	17.26
